# Spatio-Temporal Dynamics of Human Intention Understanding in Temporo-Parietal Cortex: A Combined EEG/fMRI Repetition Suppression Paradigm

**DOI:** 10.1371/journal.pone.0006962

**Published:** 2009-09-11

**Authors:** Stephanie Ortigue, James C. Thompson, Raja Parasuraman, Scott T. Grafton

**Affiliations:** 1 Department of Psychology, Institute for Collaborative Biotechnologies, UCSB Brain Imaging Center, Santa Barbara, California, United States of America; 2 Department of Psychology, George Mason University, Fairfax, Virginia, United States of America; The University of Western Ontario, Canada

## Abstract

Inferring the intentions of other people from their actions recruits an inferior fronto-parietal action observation network as well as a putative social network that includes the posterior superior temporal sulcus (STS). However, the functional dynamics within and among these networks remains unclear. Here we used functional magnetic resonance imaging (fMRI) and high-density electroencephalogram (EEG), with a repetition suppression design, to assess the spatio-temporal dynamics of decoding intentions. Suppression of fMRI activity to the repetition of the same intention was observed in inferior frontal lobe, anterior intraparietal sulcus (aIPS), and right STS. EEG global field power was reduced with repeated intentions at an early (starting at 60 ms) and a later (∼330 ms) period after the onset of a hand-on-object encounter. Source localization during these two intervals involved right STS and aIPS regions highly consistent with RS effects observed with fMRI. These results reveal the dynamic involvement of temporal and parietal networks at multiple stages during the intention decoding and without a strict segregation of intention decoding between these networks.

## Introduction

Understanding the intentions of other people is a complex activity that depends on both automatic and reflective interpretations of observed actions. Because the same motor act may lead to different outcomes, the comprehension of intentions goes beyond the simple perception of a movement towards an object [1]–[3]. Decoding of intentions involves the integration of an agent's motor actions [1], [3]. In naturalistic situations, as when observing a person grasping a hairdryer, the comprehension of the action outcomes depends on the ability to rapidly integrate a dynamic flow of visual information. This extends from the kinematics of the person's hand shaping and recognition of the hairdryer to ‘higher order’ integrated representations of the meaning and intent of the action based on the hand-object interaction, such as an evaluation of whether the actor's finger is placed on the hairdryer trigger in order to use it. According to current theories of the organization of goal directed behaviors, the ability to understand intentions emerges from a cascade of decoding operations within a representational hierarchy [3], [4]. Recent studies demonstrate that understanding of intentions is also due in part to the automatic reactivation of pre-stored templates that have been integrated over time from one's own motor skills and life experiences, consistent with the perspective of embodied cognition [2], [3], [5]–[7].

Intention understanding is thought to recruit two functionally separable cortical networks [3], [8]. The first, referred to as the “action observation network” (AON) is located in an inferior fronto-parietal network (FPN) [3]. Convergent evidence suggests that the AON is particularly important for integrating sensori-motor information during perceptual judgments about actions [9], [10], and also for understanding hand-object interactions and intentions on the basis of embodied cognitive mechanisms [3], [9], [11], [12]. In addition to the AON, intention understanding might recruit brain areas involved in social interaction [3]. This network, now referred to as the “social-network” (SN) includes the medial prefrontal cortex, precuneate cortex, insula and amygdala [3]. Both networks include the superior temporal sulcus (STS), and the region that goes beyond the fundus and the banks of STS; the superior temporal gyrus, the middle temporal gyrus (MTG), the part of the angular gyrus that is near the ascending limb of STS [13]–[17]. Interestingly, the STS region, notably its posterior part, is recruited by relatively low level processes such as observation of biological motion [18], and also higher level operations such as social inferential processing in tasks requiring mentalizing, animacy detection and theory of mind [19]–[21].

Because of the multifunctional role of the temporal cortex areas involved in both the AON and SN, ongoing debates continue over the degree to which these networks are functionally dissociable and whether they temporally interact with one another [3]. There is parallel debate on the extent to which FPN may be activated with or without activation of the STS regions [9], [22], [23]. It is not clear whether this putative dual-system model of action decoding operates in serial or parallel and the degree to which recruitment in these areas is based on automatic or reflective inference about an action.

To date, the poor temporal resolution of fMRI neuroimaging has limited the characterization of the temporal dynamics of intention decoding both within and between these two functional networks [1], [3], [4], [11], [21], [24]. Methods using millisecond temporal resolution, e.g., direct electrophysiological recordings, surface EEG recordings, or magneto-encephalography hold promise to unravel the temporal dynamics of action observation and intention understanding [25], [26]. For example, using magneto-encephalography during the imitation of lip movements, Nishitani and Hari (2000) revealed a flow of information from components of the SN and the AON network over time [27]. However, because this was an imitation task, it is unclear whether the shift into FPN was a part of action decoding, working memory or motor preparation. More recently, Van der Cruyssen et al. tried to specify the temporal dynamics of decoding action intent by performing an event related potential study where participants had to decode action intentions based on the reading of the last word of sentences describing the behavior of an agent, and from which a specific intention could be inferred [26]. Because this task was driven by verbal cues, it remains unclear if the findings would generalize to the decoding of actions observed visually. Nevertheless, together, these previous studies demonstrate that it is possible to characterize the dynamics of action understanding across cortical regions and that much of the information processing in these two networks occurs within the first 400 ms. However, the latency of brain recruitment relative to the action-related stimulus (and therefore at which processing stage modulation of a brain network occurs) remains unclear from this prior work. Similarly, the latency at which brain areas within FPN and STS regions emerge with respect to one another during intention decoding remains unknown.

**Figure 1:**
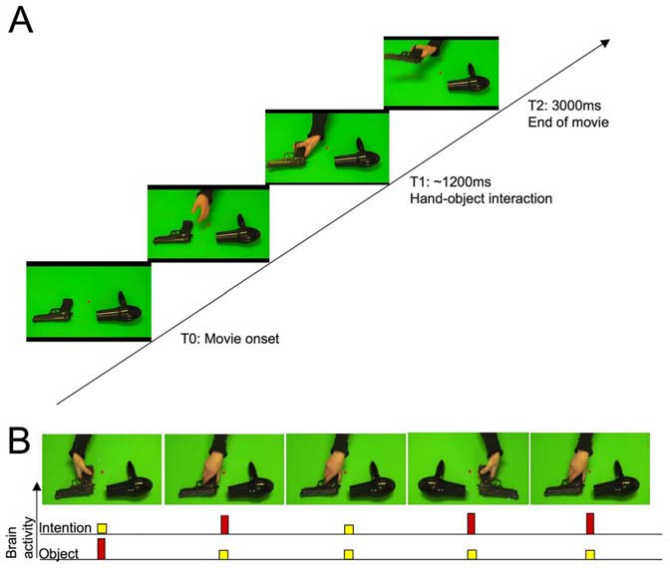
Stimulus sequence and paradigm. A. *Sequence of stimuli presentation*. Every trial consisted of the following sequence: T0: movie onset, T1: hand-object interaction followed by T2: end of movie. The example reported here represents a sample of an agent grasping a gun to use it. Task instruction was the following: “In this experiment, you will be asked to look at a centred red square during the entire session while trying to guess the intention of the actor in every video clip once the hand encounters the object (e.g., to use the object or to transport it).” B. Repetition suppression paradigm. Stimuli were presented in a mixed event-related/block design, with repetition suppression (RS) effects of intention (to transport vs. to use) and object (gun vs. hairdryer) examined within blocks. The example reported here represents a sample of an RS effect (in yellow for suppression and red for activation) that may occur on object goal (i.e., the use or WHAT) or action goal (i.e., the intention or WHY).

To characterize temporal dynamics in the FPN and STS regions further, we conducted a motor intention inference task (IIT; an exemplar of IIT is displayed on Figure 1) combined with high-density visual event-related potentials (VEPs) and fMRI recordings from 24 healthy human individuals. During the IIT task, participants were instructed to attend to a series of 3 s-video-clips displaying natural hand-on-object actions. Participants were asked to try to decode, as quickly as possible, “why” actions were being performed (e.g., to use or to move one of two objects). More precisely, participants were required to respond within 1000 ms after hand-object interaction, forcing them to make rapid judgments with minimal reflective thought. In this way we could focus on the degree to which intention decoding emerges by bottom-up processing.

As a diagnostic measure, we used repetition suppression (RS) in order to avoid the many confounds that commonly emerge from direct cognitive subtraction between different types of tasks, such as the decoding of meaningful versus meaningless actions [25]. While RS has been used in numerous prior fMRI studies [1], [4], [28]–[31], to our knowledge, the present study constitutes the first RS EEG study on the spatio-temporal dynamics of intention understanding.

The key hypothesis of the current experiment was that both the FPN and posterior temporal cortical regions (including STS) are recruited automatically, and thus early in the course of intention understanding. Specificity for this early recruitment would be based on the sensitivity of both fMRI and event-related potential measurements to RS at the level of what was being intended by an agent and not by the specific object that was being grasped.

## Results

### Behavioral results

#### Accuracy

**Figure 2:**
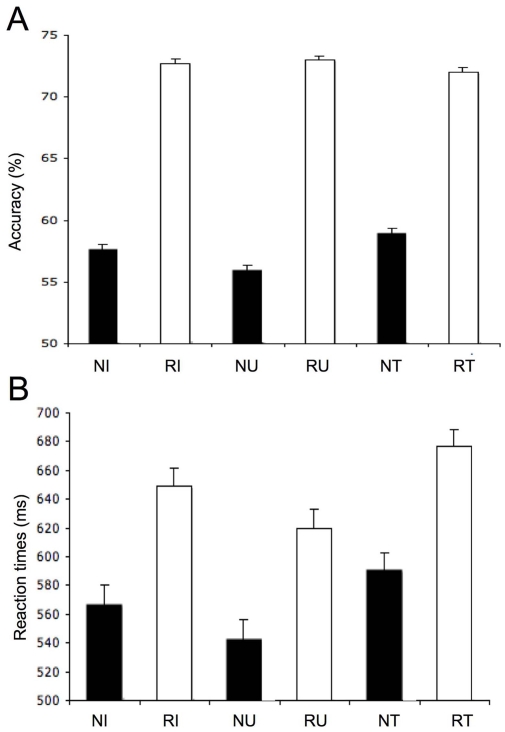
Behavioral results for intentions. A. Accuracy. Percentages of accuracy are shown for every type of intentions (NI: new intentions; RI: repeated intentions; NU: new utilization; RU: repeated utilization; NT: new transport; RT: repeated transport). Accuracy was higher for repeated intentions (72%) than for new intentions (58%). Although no main effect of intention type or object was observed (*P*>0.05), a significant interaction was observed between intention and object types (F(1,23) = 111.12). A significant interaction was also observed between intention and object types as a function of RS effects (F(1,23) = 33.3; *P*<0.0001), suggesting that stimuli were better recognized when both intentions were repeated as opposed to either being new. Results are reported at the P<0.001 level. B. Reaction times. Reaction times (in millisecond) are shown for every type of intentions (NI: new intentions; RI: repeated intentions; NU: new utilization; RU: repeated utilization; NT: new transport; RT: repeated transport). A significant main RS effect was observed for intentions across types (F(1,23) = 18.75; P<0.0004), suggesting that new intentions (567 ms) are faster recognized than repeated intentions (649 ms). Results are reported at the P<0.001 level.

No main effect of judging the type of intention was observed (*P *>0.05). On the other hand, a significant interaction was observed between intention and object types (F(1,23) = 111.12; *P *<0.0001), suggesting that the stimuli are better recognized when they are meant to be used as guns that can be fired or when they are meant to be transported as hairdryers (Table S1). In line with classic RS effects, the present RS paradigm revealed higher accuracy rates for repeated intentions (72%) than for new intentions (58%; F(1,23) = 213.86; *P *<0.0001; Figure 2A, Table 1). The high error rates confirm that participants had minimal time for explicit analysis before responding.

**Table 1:** Reaction times and accuracy: performance measures across types of intentions (new vs. repeated).

	Reaction times		% Accuracy	
	Mean (ms)	(S.E.)	Mean (%)	(S.E.)
Intention type				
**Utilization**	**582**		**65**	
New	543	(13.36)	56	(.38)
Repeated	620	(13.28)	73	(.33)
**Transport**	**634**		**66**	
New	591	(12.02)	59	(.39)
Repeated	677	(11.51)	72	(.35)
**New**	**567**		**58**	
**Repeated**	**649**		**72**	

All ANOVAs had a level of significance set to 0.05.

#### Reaction times

A main effect of intention type was observed F(1,23) = 38.05; P<0.0001), suggesting that “utilization” intentions (582 ms) were faster recognized than “transport” intentions (634 ms; Table 1). A significant main RS effect was observed for intentions across types (F(1,23) = 18.75; P<0.0004), suggesting that new intentions (567 ms) are recognized faster than repeated intentions (649 ms). The reaction time results establish that participants were well engaged in the task. The pattern of reaction time results rules out the possibility that the fMRI and EEG RS effects were due to decreasing time spent processing the stimuli on task (and shorter reaction times) for repeated items.

### NeuroImaging Results for New versus Repeated Intentions

#### Functional MRI results

**Figure 3:**
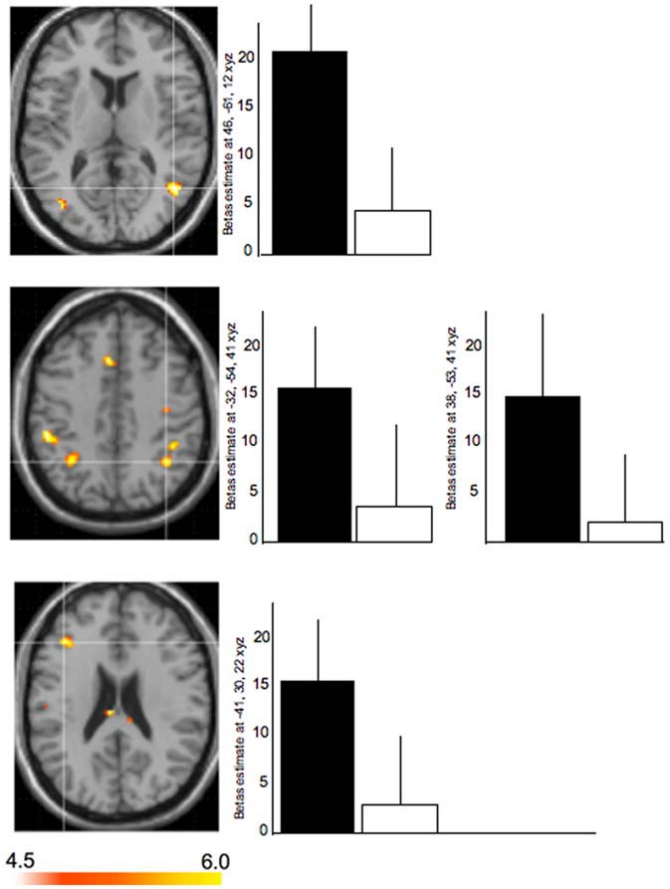
fMRI neuroimaging results for intentions. Functional MRI results when participants saw repeated actions with the same intentions (action goals). RS was observed in the following areas. Top panel: right MTG/STS (46,−61,12 x, y, z MNI coordinates; Z: 4.12), Middle panel: bilateral aIPS (left: −32,−54,41 x, y, z MNI coordinates; Z value: 3.3; right: 38,−53,41 x, y, z MNI coordinates; Z value: 3.45). Bottom panel: left inferior frontal gyrus (−41,30,22 x, y, z MNI coordinates; Z value: 3.47). Weaker areas are reported in Table 2. BOLD responses are shown on axial views.

Functional MRI results revealed significant RS in bilateral aIPS, right STS, inferior parietal lobule, and left inferior frontal gyrus when participants saw new intentions versus repeated intentions (Figure 3; Table 2). Other weaker activations were present in the superior and medial frontal gyrus, and left temporo-occipital region (Table 2). These regions did not show significant RS effects for the specific object that was grasped, (Supporting Information S1; Table S2). These findings establish the sensitivity and specificity of both posterior temporal and FPN to action decoding at the level of intentions.

**Table 2:** Local Maxima in MNI coordinates of cerebral activations peaks from fMRI data for new intentions minus repeated intentions.

Brain region labels	x	y	z	Z	Cluster size
MTG/STS	46	−61	12	4.12	104
IPL	−50	−32	45	3.88	67
	42	−41	45	3.38	49
IPL/aIPS	38	−53	41	3.45	50
	−32	−54	41	3.3	101
Temporo-occipital cortex	−38	−74	11	3.74	29
Superior frontal gyrus	−3	15	49	3.70	162
Inferior frontal gyrus	−41	30	22	3.47	53
Superior frontal sulcus	−34	−14	55	3.45	61
IFG	−50	6	30	3.2	18

Local maxima, with a Z values greater than 3 in each cluster, are provided in the table. Cluster size is in voxels. When the cluster encompasses more than one anatomical location, the localization given corresponds to the local maxima with the highest value. P<0.005 uncorrected.

#### EEG neuroimaging results

**Figure 4:**
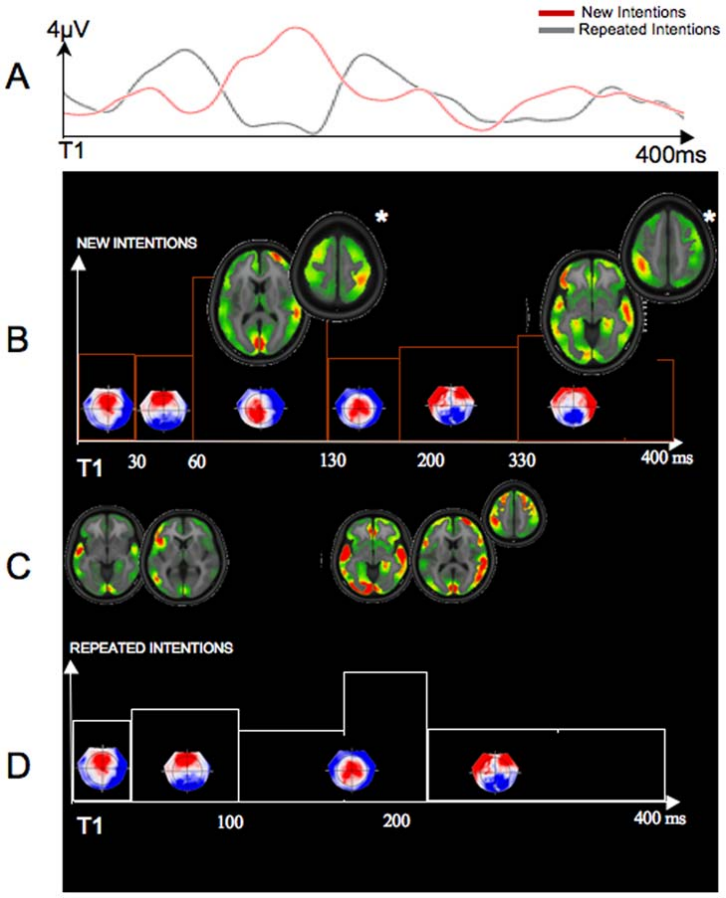
Electrical neuroimaging results for intentions. A. Schematic representation of the global field power for new intentions (NI; in red) and repeated intentions (RI; in black) from T1 to 400 ms post-T1. B. The microstate analysis identified a total of six time periods of stability for NI. NI-Map 1: 0–30 ms; NI-Map 2: 32–60 ms; NI-Map 3: 62–130 ms; NI-Map 3: 132–200 ms; NI-Map 4: 202–330 ms; NI-Map 4: 332–400 ms. For the RI, only four time periods of stability were detected, i.e., RI-map 1: 0–30 ms; RI-Map 2: 32–100 ms; RI-Map 3: 102–240 ms; RI-Map 4: 242–400 ms. The main differences between NI and RI conditions were observed at two different time intervals: i) between 62 and 130 ms; and ii) between 332 and 400 ms post hand-on-object interaction. Here, maps are represented on top of schematic representation of GFP. All topographies are shown with the nasion upward and left scalp leftward. LAURA source estimations of these two intervals were characterized by right-lateralized activations including right STS (local maximum: right posterior STS: 60,−37,7; x, y, z mm Talairach coordinates) and bilateral aIPS (local maximum: right aIPS: 39,−37,54; x, y, z mm Talairach coordinates) for the first interval, and the recruitment of a distributed brain network, mostly in the left aIPS (local maximum: −40,−43,48; x, y, z mm Talairach coordinates), right MTG/STS (local maximum: −53,−55,17; x, y, z mm Talairach coordinates), left IFG (local maximum: −48,33,−3; x, y, z mm Talairach coordinates) for the second interval. Differential activations between NI (B) and RI (D) are represented on top of the schematic representation of the GFP. C. Common LAURA brain source estimations for both NI and RI conditions are shown here. These brain source estimations were localized within anterior MTG/STS, bilateral occipital region, temporo-occipital region, and anterior cingulate.

High-density EEG neuroimaging, combining brain microstate analysis with Local Auto-Regressive Average (LAURA) distributed linear source estimations, expanded these fMRI results by revealing the temporal dynamics of new intentions as compared to repeated intentions (Figure 4A–D). For the new intention (NI) condition, the brain microstate analysis revealed a total of six time periods of stability (i.e., New Intention-Map (NI-Map) 1: 0–30 ms; NI-Map 2: 32–60 ms; NI-Map 3: 62–130 ms; NI-Map 3: 132–200 ms; NI-Map 4: 202–330 ms; NI-Map 4: 332–400 ms; Figure 4B). For the repeated intention (RI) condition (Figure 4A in red, Figure 4B), only four time periods of stability were detected (i.e., Repeated Intention-Map (RI-map) 1: 0–30 ms; RI-Map 2: 32–100 ms; RI-Map 3: 102–240 ms; RI-Map 4: 242–400 ms; Figure 4D). The main differences between the NI and RI conditions were observed at two different time periods after hand-on-object interaction: the first occurred between 62 and 130 ms; the second difference occurred between 332 and 400 ms post hand-on-object interaction (Figure 4). The first temporal difference (between 62 and 130 ms) was reinforced by a significant global field power (GFP) difference between NI (in red in Figure 4A) and RI (in black in Figure 4A) during this time period. More precisely, the GFP peaked at 126 ms for novel intentions, although group-averaged GFP for the repeated intention condition was almost extinguished at the same time period (P<0.05). The reliability of this difference observed at the group-averaged level was confirmed at the individual level using a Bonferroni corrected paired t-test on GFP (significant greater GFP difference for novel intentions in comparison with repeated intentions from 118 ms to 134 ms, with a peak at 126 ms; t = 2.71; P = 0.013). No GFP difference was observed for the second time period, suggesting that, at this time, the two conditions varied as a function of topography rather than power. LAURA source estimations of the topography of these two significant periods of stability revealed similarities with our fMRI data by showing a distributed network mostly characterized by right-lateralized activations including right STS (local maximum: right posterior STS: 60, −37, 7; x, y, z mm Talairach coordinates) and bilateral aIPS (local maximum: right aIPS: 39, −37, 54; x, y, z mm Talairach coordinates; Figure 4B) for the first significant time period, and the recruitment of a distributed brain network, mostly in the left aIPS (local maximum: −40, −43, 48; x, y, z mm Talairach coordinates), right MTG/STS (local maximum: −53, −55, 17; x, y, z mm Talairach coordinates), left inferior frontal gyrus (local maximum: −48, 33, −3; x, y, z mm Talairach coordinates) for the second time period (Figure 4B; Table 3). Other weaker activations are reported in Table 3. Outside of these two time windows of difference, LAURA brain source estimation revealed similar topographies and brain source estimations for both the NI and RI conditions (Figure 4C). These brain source estimations were localized within anterior MTG/STS, bilateral occipital region, temporal occipital cortex, and anterior cingulate (Table 3). Microstate analysis for novel versus repeated objects demonstrated a different sequence of brain sources (including the following current source maxima: occipito-parietal, left anterior temporal lobe, anterior cingulate, right inferior frontal gyrus (IFG), and right anterior temporal lobe; see Supporting Information S1; Table S3, Figure S1 and Figure S2), confirming that the RS differences for intention decoding described above were both temporally and spatially specific.

**Table 3:** Local maxima of current source density obtained from LAURA brain source estimations of EEG data for new and repeated intentions.

Microstate time periods for new intentions	Microstate time periods for repeated intentions	Brain region labels	x	y	z
0–30 ms	0–30 ms	left MTG	−53	−2	8
32–60 ms	32–100 ms	Left inferior frontal gyrus	−42	27	1
62–130 ms^*^	-	**Right STS region**	**60**	**−37**	**7**
		**Right aIPS**	**39**	**−37**	**54**
		**Occipital lobe**	**3**	**−82**	**6**
		**Right medial frontal gyrus**	**27**	**55**	**13**
		**Anterior cingulated**	**3**	**35**	**−10**
		**Left MTG/STS**	**−48**	**−63**	**15**
		**Left MTG**	**−58**	**−11**	**−9**
132–200 ms	102–240 ms	Left MTG	−53	−11	−9
202–330 ms	242–400 ms	Right MTG	53	−6	−9
332–400 ms^*^	**-**	**Left IPL/aIPS**	**−40**	**−43**	**48**
		**Right STS region**	**53**	**−55**	**17**
		**Right MTG**	**60**	**−26**	**−6**
		**Left MTG**	**−53**	**−6**	**−9**
		**Left IFG**	**−48**	**33**	**−3**

Local maxima (in Talairach coordinates) of periods of brain stability (i.e., microstates) are provided in the table. Asterisks indicate time periods that are significantly present in the novel intention condition in comparison with the repeated intention condition. P<0.05.

## Discussion

The ability by fMRI to distinguish dual systems for intention understanding, i.e., one distributed across fronto-parietal cortex and incorporating the putative human mirror neuron system [9], [32] and another more broadly distributed “social network” has led to strong arguments about their relative importance, functional independence and specificity [24], [33]. On the one hand, it has been argued that the AON is sufficient for decoding all intentions and more generally social reasoning via mirror neuron mechanisms [9]. On the other hand, a strong case has been made that processing related to inference is conducted within the SN alone [20], [21]. By combining event-related fMRI and high-density EEG recordings with a repetition suppression approach, here we could address this problem more directly and test for evidence of automatic early recruitment across these networks in an intention understanding task.

### Behavioral performance and intention understanding

In line with prior behavioral studies of adaptation, repeated intentions were identified more accurately (72%) than new intentions (58%). These behavioral findings confirm the existence of different neural processes between new and repeated trials during intention understanding. A significant adaptation effect was also observed for speed of decoding intentions as ascertained by reaction times, which showed faster intention decision making for new (567 ms) versus repeated intentions (649 ms). This opposite pattern of behavioural performance (i.e., slower reaction times for decoding repeated intentions) is in line with previous work demonstrating that behavioural attenuation effects (rather than priming effects) may occur as a function of the task instruction [34]. Also, the pattern of reaction time results rules out the possibility that the fMRI and EEG RS effects were due to decreasing time spent processing the stimuli on task (and shorter reaction times) for repeated items. Interestingly previous studies have shown that behavioural adaptation may reflect stimulus-specific processing independent of task demands rather than a reduced processing time [34]. In the present study, this suggests that a specific neural network implying automatic analysis of repeated versus new information may be engaged during intention understanding.

### fMRI localization for intention understanding

By using RS with fMRI we identified a specific subset of brain regions within both the human AON and SN that are specifically sensitive to intention understanding. Despite the uncertainties about the neural mechanisms underlying RS, i.e., “fMRI adaptation” [28], [34]–[37], our data are in line with the principle that the number of neurons that are important for stimulus representation and processing remain constant but show reductions in their firing rates or duration of firing for repeated stimuli [28], [36]. In agreement, the present fMRI RS results revealed a systematic increase of activation in the right MTG/STS, inferior parietal lobule (IPL), aIPS, and inferior frontal gyrus (IFG) in response to the presentation of new intentions as opposed to repeated intentions. The occurrence of RS effects in heteromodal cortex reinforces the evidence that RS occurs across the neocortex and is not limited to only low level areas such as the visual cortex. This highlights the growing potential of using stimulus-specific RS effects to study cognitive operations beyond basic perceptual features. In our data, the specific functional dynamic for decoding intentions was different than that for decoding lower level visual features related to the specific object that was grasped (Supporting Information S1). The network for understanding intentions extends beyond brain areas involved in object shape, size, and orientation (e.g., ventral visual areas).

The present findings reinforce previous work showing a recruitment of IPL and IPS for hand action observation and goal representation [18], [21], [38], [39]. Current human data reinforce a growing body of studies which demonstrate a strong role of aIPS in goal and outcome understanding and so challenge the classic view suggesting that aIPS acts as a repository of grip apertures generated from object features [3].

Another key finding from the current fMRI RS results is the sensitivity to intention in the STS. This reinforces the critical role of the STS in action interpretation [18], [21]. The current fMRI task design and temporal resolution does not allow us to make any conclusions whether this STS sensitivity involves more complex inferential processes such as mentalizing or theory of mind [19]–[21]. However, the results of the present study suggest this region, in conjunction with aIPS, might be involved in decoding intentions at the earliest stages of action recognition.

The involvement of the inferior frontal gyrus, a brain region involved in cognitive functions such as planning and performing action, reinforces recent evidence demonstrating a crucial role of this brain area in intention understanding [11], [40]. In support of this, lesions circumscribed to IFG have been associated with deficits in matching pictures of actions with appropriate objects. Also, recent virtual lesion studies based on transcranial magnetic stimulations to IFG in healthy subjects demonstrate interference by means of increased response times in matching tasks that involve body actions.

### Temporal dynamics of intention understanding

Our EEG findings identify modulation in the temporal dynamics of intention understanding by demonstrating two time intervals with sensitivity to RS across the collective 400 ms after hand-object interaction onset. The present data are also in line with previous work showing early and/or late repetition effects as indexed with visual event-related potentials (VEPs). Previous work has also demonstrated that RS effects for higher-order cognitive mechanisms can emerge as early as 75 ms after stimulus onset and then around 400 ms [37], [41]. Early and late RS effects have also been described in studies of object vision [37]. Our results identify an early RS effect between 62 and 130 ms after grip onset; and also a later one around 330 ms. This very early change in the GFP shows that when observing actions done by others, individuals may automatically begin to understand the *intention* of an action (e.g., to hold a gun to shoot) in an early stage of visual processing, i.e., after only 62–130 ms.

The evidence of early differences in visual processing for action intentions after hand-object interaction suggests participants are generating automatic judgments about observed intentions. This is further supported by our behavioral results. The accuracy rates were low during imaging (e.g., around 60% for novel intentions) due to the temporal constraint imposed in the IIT task. This interpretation that participants only had time for rapid judgments is further supported by the high accuracy rate (100%) we obtained from the participants when they had no time constraints during the debriefing session. i.e., when participants had time to make explicit and slower inferential judgments on intentions (See Supporting Information S1).

### Spatiotemporal Dynamics of Intention Understanding

Source estimations of the RS sensitivity to intention support a two stage-process model by showing modulation of activity in different cortical networks as a function of time. Critically, these estimations show a significant recruitment within right STS and bilateral aIPS in the early stage of information processing, beginning shortly after the start of the hand-object interaction. The EEG sources for these early RS effects as indexed by VEPs are anatomically consistent with the localization of RS effects obtained in our BOLD fMRI analysis. In both cases the areas are distinct from those for RS of object or other lower kinematic features. This supports a relative hierarchical model of action understanding, with nested levels of information processing distributed across cortex [3], [42]. The presence of recruitment in STS and aIPS early in visual processing data are also in line with recent electrophysiological evidence from both animal and human studies arguing for a bidirectional mechanism where these associative areas can be recruited in very early stages of information processing (∼60 ms) [43]. Along these lines, the early visual evoked potentials (VEPs) in associative areas in our study may not be exclusively generated by sensory activation but might also be generated by input from higher-order areas amplifying the decoding of intention relevant features. A later stage of intention specific information processing was observed in a more widely distributed posterior right temporal region and left aIPS network, with an additional involvement of left inferior frontal gyrus.

Together, the intention specific adaptation observed at these two stages indicates that right STS and aIPS regions do not simply function as part of a serial, unidirectional network. Instead, our findings suggest the repeated involvement of STS and aIPS as observers engage in the process of decoding the intentions of other people. The spatio-temporal dynamics of intention decoding provided by the EEG and source localization methods used in the present study substantially supplement the results observed in the fMRI RS data. While the pattern of intention specific fMRI RS is remarkably consistent with the source estimates of RS EEG, the former presents only a static picture of what is a highly dynamic process.

## Methods

### Population

Twenty-four men ranging from 19–42 yrs in age participated in the present study. All were right-handed with normal (or corrected) vision, and no psychiatric or neurological diseases, as ascertained with a detailed anamnesis. All participants gave their informed written consent to take part in the study that has been approved by the University of California Santa Barbara's Institutional Review Board.

### Procedure

We used a repetition suppression paradigm in an event-related experiment. Participants were asked to perform a motor intention inference task while the experimenter monitored their performance on a computer. This motor intention inference task was performed both in EEG and fMRI, i.e., two sessions a day apart from one another.

### Stimuli material

A total of 32 sets of 3 second-long action video clips were generated. Each video clip depicted a hand reaching out to either transport or use a hairdryer or a gun (e.g., Figure 1; video S1, video S2). Every video clip consisted of the following sequence: T0: movie onset, T1: hand-object interaction followed by T2: end of movie. T0 consisted in an establishing scene with one object (a gun or a hairdryer) on a green background. The gun and the hairdryer were placed on the green background at equal distance on either side of the center of the video frame. Video clips were recorded with non-directional lighting against a green background to enable later editing of the stimuli with video software. The video camera was positioned on a tripod and angled downward to capture the arm and hand reaching for the target from a third person perspective (approx. 45 degrees from the horizontal plane of the desktop). All video clips were created by filming intentional natural actions on an object. Five hundred milliseconds after T0, the actor's hand was required to approach the object with a controlled speed and controlled hand aperture that was similar for every condition. At T1, i.e., after about 1200 ms after T0, the actor's hand grasped the object. Two types of intentions were manipulated: To use or to move an object. The position of the object (right visual field, left visual field), the target object (gun, hairdryer), the acting hand (right, left), the direction the object was facing (right, left) and the kinematics of the grip (overhand, underhand) were controlled and alternated over 32 video clips. The conjunction of acting hand, direction the object was facing, and kinematics of the grip determined if an action reflected a utilization grasp (e.g., right hand + underhand + left-facing object) or a transport grasp (e.g., right hand + underhand + right-facing object). The wide variety of stimuli meant that we could be confident that the results revealed a general neural representation of intention understanding, rather than being an epiphenomenon of one particular type of video clip. Each reach to grasp was completed and edited to exactly 3-second segments using video editing software Adobe Premiere Pro 2.0 (Adobe Systems, San Jose, CA). T2 corresponded to the end of T1 (Figure 1). Reaction times were collected from T1.

### Task Instruction

“In this experiment, you will be asked to look at a centred red square during the entire session while trying to guess, as quick as possible, the intention of the actor in every video clip once the hand encounters the object (e.g., to use the object or to move it).”

### Experimental design

Participants viewed sequences of video clips separated by a blank screen. In order to avoid any saccadic movements, a fixation red square remained constantly on the screen (Figure 1A). After a sequence of eight video clips, a 6 s-long screen reminding the task instruction was systematically presented. Each sequence of eight movies began with a randomly chosen video clip designated as being novel. The subsequent video clips were chosen among the remaining set of video clips according to a pseudo-random order. Novel and repeated trials were defined in relation to the previous trial only (Figure 1B). Each participant completed five runs with 8 sequences in each run, giving a total of 64 trials per run. Experimental blocks were intermixed for every participant. The same event-related design was used in both fMRI and EEG session. Inter-trial interval varied in 2000 ms random increments from 2000 to 6000 ms.

### Repetition Suppression Procedure

Stimuli were presented in a mixed event-related/block design, with repetition suppression (RS) effects of intention (to move vs. to use) and object (gun vs. hairdryer) examined within blocks. In this IIT, the stimuli were arranged so that different aspects of the observed motor action were repeated from trial to trial. In parallel we tested for concomitant suppression of brain activity [28], [44]. More precisely, in the current experiment, the position of the two objects, which object was grasped, how the objects were grasped (underhand or overhand grip) and why it was grasped (to transport or to use) were independently manipulated. The two objects in each trial were closely paired in terms of size, color, and shape, so that grasp configuration was similar (Figure 1). In this way, repetition of intention could be separated from other kinematic aspects of hand-object interactions. The order of presentation of each block was randomized across participants. Within each block, the trial order of stimulus repetition was pseudo-randomized by initially randomly drawing the first video-clip to be presented as novel. The order of the eight movies that followed came from one of eight pre-determined trial sequences that ensured two repetitions of each combination of object (gun, hairdryer) and intention (transport, utilization) within each block. This RS procedure allows to characterize the temporal dynamics within brain networks that are sensitive to the intentions of an action and distinguish these from lower level stimulus features [3].

### Data acquisition

Both functional (fMRI) and electrical (VEPs) neuroimaging were conducted at the University California Santa Barbara Brain Imaging Center.

#### fMRI data acquisition

Functional MRI recordings were conducted using a 3T TIM Trio Siemens Magnetom with a 12 channel phased-array head coil. Foam padding was used for head stabilization. For each functional run, an echo planar gradient-echo imaging sequence sensitive to BOLD contrast was used to acquire 33 slices per repetition time (TR) (3 mm thickness), with a TR of 2000 ms, echo time of 30 ms, flip angle of 90 degrees, field of view of 192 mm, and 64×64 matrix. Before all the functional runs, a high-resolution T1-weighted sagittal sequence image of the whole brain was acquired (TR = 15.0 ms; echo time = 4.2 ms; flip angle = 9 degrees, 3-D acquisition, field of view = 256 mm; slice thickness = 0.89 mm, matrix = 256×256).

#### VEP data acquisition

Continuous electroencephalogram (EEG) was recorded from 128 AgCl carbon-fiber coated electrodes using an Electric Geodesic Sensor Net® (GSN300; Electrical Geodesic Inc., Oregon; http://www.egi.com/), where EEG electrodes are arrayed in a regular distribution across the head surface and the inter sensor distance is approximately 3 cm. The EEG was sampled at a rate of 500 Hz, and band-pass filtered at 0.01–200 Hz with the vertex electrode (Cz) serving as an on-line recording reference. Impedance was kept below 50 kΩ.

### Data analysis

#### fMRI data analyses

Functional MRI analysis was carried out in SPM5 (www.fil.ion.ucl.ac.uk/spm). Data were realigned to correct for head movements. All realigned functional images were registered to the anatomical image. The anatomical images were then transformed to conform the Montreal National Institute (MNI) space and the parameters of this transformation were applied to the functional data and smoothed with an 8-mm full width half maximum filter. A design matrix was fitted for each subject with the trials in each cell of the 2×2×2×2×2×2 factorial design (intention types (to use vs. to move); objects (gun vs. hairdryer); presentation types (new vs. repeated); position of object (left visual field vs. right visual field); kinematics of the grip (under vs. over), hand of action (left vs. right)), modeled by a standard hemodynamic response function and its temporal derivative. Each trial was modeled as a single event, starting at the onset of the hand-on-object interaction. Rest was not modeled. The design matrix was fit to the data for each participant individually. After estimation, betas were taken to the second level for random effect analysis to identify which brain areas were preferentially activated novel intentions in comparison with repeated intentions (novel intentions > repeated intentions). Anatomical labeling was ascertained by the probabilistic brain atlas from the Laboratory Of NeuroImaging at the University of California Los Angeles. This atlas provides a series of maps of brain anatomic regions, that were produced from a set of whole-head MRI of 40 human volunteers, and which includes a set of 56 structures in the brain.

#### VEP data analyses

Continuous electroencephalogram (EEG) data were imported, averaged and analyzed in Cartool (version 3.32). Epochs of analysis were visually inspected for oculomotor (saccades, and blinks), muscles, and other artifacts in addition to an automated threshold rejection criterion of 100 µV. After off-line artifact rejections, VEPs were computed covering 400 ms after the onset of hand-on-object interaction. This 400 ms time window of analysis was based on previous studies that showed temporal modulations of action and intention decoding within the first 400 ms of information processing [40]. VEP data were then baseline corrected, and band-pass filtered between 1 and 30 Hz. VEP data were then recalculated off-line against the average reference, and normalized to their mean global field power (i.e., GFP) before group averaging. The GFP, computed as the spatial standard deviation of the scalp electric field, yields larger values for stronger electric fields and is calculated as the square root of the mean of the squared value recorded at each electrode (vs. the average reference).

#### Microstate analysis

VEPs data from novel and repeated intentions were then submitted to a brain microstate analysis in order to detect every period of brain state stability (microstate) after hand-on-object interaction [45]. To identify start and end of each optimal microstate, a standard cluster analysis was employed using the grand-mean VEPs of each condition [45], [46]. This cluster analysis uses a hierarchical agglomerative cluster-algorithm to identify the predominant topographies (i.e., maps) and their sequence within a data set. The optimal number of maps (i.e., the minimal number of maps that best accounts for the data set) is determined based on a modified Krzanowski-Lai criterion [47]. Importantly, this cluster analysis is reference-free, and insensitive to amplitude modulation of the same scalp potential field across conditions, since normalized maps are compared. It was performed across time and experimental conditions in order to determine whether and when novel versus repeated intentions engaged distinct configurations of intracranial generators. Then, the pattern of maps observed in the group-averaged data was statistically tested at the individual level using a competitive fitting procedure that determines whether a given experimental condition is more often described by one map versus another. Durations of every period of brain stability (microstate) were subjected to a repeated measure ANOVA. Results were accepted as significant at P<0.05. GFP were also statistically tested at the individual level using a Bonferroni corrected paired-test.

#### Brain source EEG analysis (Local Auto-Regressive Average, LAURA)

An intracranial brain source analysis was calculated for each stable period of time (microstate) found between 0 and 400 ms using the Local Auto-Regressive Average (LAURA) model of the unknown current density in the brain [48]. LAURA was implemented using a lead field (solution space) calculated on a realistic head model including 3005 solution points selected from a 6×6×6 mm grid equally distributed within the gray matter. Source estimations were rendered on the MNI/McGill average standard brain as supplied by Cartool. Then transformation between the MNI coordinate system and that of Talairach and Tournoux was performed with Cartool.

## Supporting Information

Supporting Information S1 (0.04 MB DOC)

Figure S1 Electrical Neuroimaging results at T0. High-density EEG neuroimaging, combining brain microstate analysis with LAURA distributed linear source localizations at movie onset (T0; A), revealed archetypal VEPs components (e.g., C1, P1 and N1). The topographic pattern analysis of the global field power (B) identified three selective time periods of stable topography (C) in the across the collective 300 ms post-stimulus period from the two conditions of interest (new in black vs. repeated in red). All topographies are shown with the nasion upward and left scalp leftward. Intracranial brain generators as estimated with LAURA inverse solution over this period of time revealed a bilateral occipito-temporal activity as shown on axial plane.
(9.70 MB TIF)

Figure S2 Electrical Neuroimaging results at T1 for new objects (A) and repeated objects (B). High-density EEG neuroimaging, combining brain microstate analysis with LAURA distributed linear source localizations applied at T1 (i.e., at the moment of hand-object interaction), revealed seven time periods of stability: Map 1: 0–30 ms; Map 2: 32–60 ms; Map 3: 62–80 ms; Map 4: 82–120 ms; Map 5: 122–150 ms; Map 6: 152–320 ms; Map 7: 322–400 ms. Three topographies (Maps 3–5) were significantly present in the novel objects condition only. Here, maps are represented on top of schematic representation of global field power. All topographies are shown with the nasion upward and left scalp leftward. LAURA source estimations of this brain topography demonstrated similarities between our VEP and fMRI data by showing a dominant anterior cingulate activation for Map 3 (local maximum: 3, 41, −9; x, y, z mm Talairach coordinates); a dominant right-lateralized activation in the right STS region and right aIPS (local maximum: 57, −11, −5; x, y, z mm Talairach coordinates) for Map 4; and a right-lateralized activation including right IFG and medial FG (local maximum: 46, 14, 25; x, y, z mm Talairach coordinates) for Map 5. Map 3's temporal window (62–80 ms after hand-object interaction) was also characterized by a significant difference of GFP between new objects and repeated objects. Group-averaged data revealed a GFP peak at 64 ms for new objects, although GFP for repeated objects was almost extinguished at the same time period. Between 80–150 ms post hand-object interaction, no significant difference was observed in terms of GFP. Interestingly, after 150 ms post hand-object interaction, the reverse pattern (greater GFP for repeated objects than suppressed objects) was observed. The reliability of this microstate at the group-averaged level was confirmed at the individual level using a Bonferroni corrected paired Ttest on GFP (P<0.05). Here, activations are represented on axial cross sections.
(8.26 MB TIF)

Table S1 All ANOVAs had a level of significance set to 0.05.
(0.04 MB DOC)

Table S2 Local maxima, with a Z values greater than 3 in each cluster, are provided in the table. Cluster size is in voxels. When the cluster encompasses more than one anatomical location, the localization given corresponds to the local maxima with the highest value. P<0.001 uncorrected.
(0.03 MB DOC)

Table S3 Local maxima (in Talairach coordinates) of periods of brain stability (i.e., microstates) are provided in the table. Asterisk indicates the time period that is significantly present in the novel object condition in comparison with the repeated object condition. P<0.05.
(0.04 MB DOC)

Video S1 Example of one of our stimuli. This video 1 shows a hand grasping a hairdryer to transport it.
(0.03 MB MOV)

Video S2 Other example of our stimuli. This video shows one hand grasping a hairdryer to use it.
(0.04 MB MOV)

## References

[1] Hamilton AF, Grafton ST (2006). Goal representation in human anterior intraparietal sulcus.. J Neurosci.

[2] Shmuelof L, Zohary E (2007). Watching others' actions: mirror representations in the parietal cortex.. Neuroscientist.

[3] Grafton ST (2009). Embodied cognition and the simulation of action to understand others.. Ann N Y Acad Sci.

[4] Hamilton AFdC, Grafton ST (2008). Action outcomes are represented in human inferior frontoparietal cortex.. Cereb Cortex.

[5] Cross ES, Hamilton AF, Grafton ST (2006). Building a motor simulation de novo: observation of dance by dancers.. Neuroimage.

[6] Aglioti S, Cesari P, Romani M, Ugesi C (2008). Action anticipation and motor resonance in elite basketball players.. Nature Neuroscience.

[7] Grafton S (2008). Malleable templates: reshaping our crystallized skills to create new outcomes.. Nat Neurosci.

[8] Keysers C, Gazzola V (2007). Integrating simulation and theory of mind: from self to social cognition.. Trends Cogn Sci (Regul Ed).

[9] Rizzolatti G, Sinigaglia C (2008). Mirrors in the brain. How our minds share actions and emotions..

[10] Wong KF, Huk AC (2008). Temporal Dynamics Underlying Perceptual Decision Making: Insights from the Interplay between an Attractor Model and Parietal Neurophysiology.. Front Neurosci.

[11] Iacoboni M, Molnar-Szakacs I, Gallese V, Buccino G, Mazziotta JC (2005). Grasping the intentions of others with one's own mirror neuron system.. PLoS Biol.

[12] Cross E, Kraemer D, Hamilton A, Kelley W, Grafton S (2008). Sensitivity of the Action Observation Network to Physical and Observational Learning.. Cerebral Cortex.

[13] Allison T, Puce A, McCarthy G (2000). Social perception from visual cues: role of the STS region.. Trends Cogn Sci.

[14] Pelphrey KA, Morris JP, McCarthy G (2004). Grasping the intentions of others: the perceived intentionality of an action influences activity in the superior temporal sulcus during social perception.. J Cogn Neurosci.

[15] Thompson JC, Clarke M, Stewart T, Puce A (2005). Configural processing of biological motion in human superior temporal sulcus.. J Neurosci.

[16] Thompson JC, Hardee JE, Panayiotou A, Crewther D, Puce A (2007). Common and distinct brain activation to viewing dynamic sequences of face and hand movements.. Neuroimage.

[17] Materna S, Dicke PW, Thier P (2008). The posterior superior temporal sulcus is involved in social communication not specific for the eyes.. Neuropsychologia.

[18] Jellema T, Baker CI, Wicker B, Perrett DI (2000). Neural representation for the perception of the intentionality of actions.. Brain Cogn.

[19] Saxe R, Xiao DK, Kovacs G, Perrett DI, Kanwisher N (2004). A region of right posterior superior temporal sulcus responds to observed intentional actions.. Neuropsychologia.

[20] Grossman ED, Battelli L, Pascual-Leone A (2005). Repetitive TMS over posterior STS disrupts perception of biological motion.. Vision Res.

[21] Brass M, Schmitt RM, Spengler S, Gergely G (2007). Investigating action understanding: inferential processes versus action simulation.. Curr Biol.

[22] Schütz-Bosbach S, Prinz W (2007). Perceptual resonance: action-induced modulation of perception.. Trends Cogn Sci (Regul Ed).

[23] Hesse MD, Sparing R, Fink GR (2008). End or Means-The “What” and “How” of Observed Intentional Actions.. J Cogn Neurosci.

[24] de Lange FP, Spronk M, Willems RM, Toni I, Bekkering H (2008). Complementary systems for understanding action intentions.. Curr Biol.

[25] Sitnikova T, Kuperberg G, Holcomb PJ (2003). Semantic integration in videos of real-world events: an electrophysiological investigation.. Psychophysiology.

[26] Van der Cruyssen L, Van Dyunslaeger M, Cortoos A, Van Overwalle F (2008). ERP time course and brain areas of spontaneous and intentional goal inferences.. Social Neuroscience.

[27] Nishitani N, Hari R (2000). Temporal dynamics of cortical representation for action.. Proc Natl Acad Sci U S A.

[28] Grill-Spector K, Henson R, Martin A (2006). Repetition and the brain: neural models of stimulus-specific effects.. Trends Cogn Sci.

[29] Ishai A, Bikle PC, Ungerleider LG (2006). Temporal dynamics of face repetition suppression.. Brain Res Bull.

[30] Dinstein I, Hasson U, Rubin N, Heeger DJ (2007). Brain areas selective for both observed and executed movements.. J Neurophysiol.

[31] Grafton ST, Hamilton AF (2007). Evidence for a distributed hierarchy of action representation in the brain.. Hum Mov Sci.

[32] Grafton ST, Arbib MA, Fadiga L, Rizzolatti G (1996). Localization of grasp representations in humans by positron emission tomography. 2. Observation compared with imagination.. Exp Brain Res.

[33] Kilner JM, Frith CD (2008). Action observation: inferring intentions without mirror neurons.. Curr Biol.

[34] Xu Y, Turk-Browne NB, Chun MM (2007). Dissociating task performance from fMRI repetition attenuation in ventral visual cortex.. J Neurosci.

[35] Wiggs CL, Martin A (1998). Properties and mechanisms of perceptual priming.. Curr Opin Neurobiol.

[36] Grill-Spector K, Malach R (2001). fMR-adaptation: a tool for studying the functional properties of human cortical neurons.. Acta Psychol (Amst).

[37] Guo C, Lawson AL, Jiang Y (2007). Distinct neural mechanisms for repetition effects of visual objects.. Neuroscience.

[38] Binkofski F, Dohle C, Posse S, Stephan KM, Hefter H (1998). Human anterior intraparietal area subserves prehension: a combined lesion and functional MRI activation study.. Neurology.

[39] Frey SH, Vinton D, Norlund R, Grafton ST (2005). Cortical topography of human anterior intraparietal cortex active during visually guided grasping.. Brain research Cognitive brain research.

[40] Pobric G, Hamilton AF (2006). Action understanding requires the left inferior frontal cortex.. Curr Biol.

[41] Pickering EC, Schweinberger SR (2003). N200, N250r, and N400 event-related brain potentials reveal three loci of repetition priming for familiar names.. J Exp Psychol Learn Mem Cogn.

[42] Hamilton AF, Grafton ST, Haggard P, Rossetti Y, Kawato M (2007). The motor hierarchy: from kinematics to goals and intentions.. Sensorimotor foundations of higher cognition.

[43] Bullier J (2001). Integrated model of visual processing.. Brain Res Brain Res Rev.

[44] Noppeney U, Penny WD (2006). Two approaches to repetition suppression.. Hum Brain Mapp.

[45] Lehmann D, Gevins AS, Re mond A (1987). Principles of spatial analysis.. Handbook of electroencephalography and clinical neurophysiology, vol 1 Methods of analysis of brain electrical and magnetic signals.

[46] Murray MM, Brunet D, Michel CM (2008). Topographic ERP analyses: a step-by-step tutorial review.. Brain Topogr.

[47] Krzanowski W, Lai YT (1985). A criterion for determining the number of groups in a data set using sum of squares clustering.. Biometrics.

[48] Grave de Peralta Menendez R, Gonzalez Andino S, Lantz G, Michel CM, Landis T (2001). Noninvasive localization of electromagnetic epileptic activity. I. Method descriptions and simulations.. Brain Topogr.

