# Supplementary material for: Spatio-Temporal Dynamics of Human Intention Understanding in Temporo-Parietal Cortex: A Combined EEG/fMRI Repetition Suppression Paradigm
Source: PLoS One. 2009 Sep 11;4(9):e6962. doi: 10.1371/journal.pone.0006962 (PMC2736621; doi:10.1371/journal.pone.0006962)
Supplement: Supporting Information S1 — (0.04 MB DOC) [file pone.0006962.s001.doc]

**Supporting Information**

**Supplementary results**

*Supplementary behavioral results*

*Novel objects versus repeated objects*

A significant main RS effect of presentation types for objects was observed (F(1,23) = 94,78; *P* < 0.0001), suggesting that repeated stimuli (objects: 70%) are better recognized than new stimuli (objects: 60%). A main effect of object types too was found (F(1,23) = 5.37; P = 0.03) suggesting that the recognition of guns (66%) is slightly better than that of hairdryers (64%). No main RS effect was observed on object reaction times (*P* > 0.05). Similarly, no main effect of object types was found (*P* > 0.05; Table S1).

*Debriefing results*

After the scanning session, participants were given a paper-pencil version of the IIT in which they had to write the agent’ intents. No time constraint was imposed. Results reached a 100% accuracy rate.

*Supplementary functional MRI results for novel objects versus repeated objects*

Functional MRI results revealed RS in right medial frontal gyrus (local maximum: 48, 17, 27, x, y, z mm, MNI coordinates; Table S2). A weaker RS effect was observed in left postcentral gyrus (local maximum: -46, -16, 30, x, y, z mm, MNI coordinates) when participants saw new versus repeated objects (Table S2).

*Supplementary EEG neuroimaging results*

At movie onset (T0):

High-density EEG neuroimaging, combining brain microstate analysis with LAURA distributed linear source localizations at movie onset (T0; A), revealed archetypal VEPs components (e.g., C1, P1 and N1). The topographic pattern analysis of the global field power (B) identified three selective time periods of stable topography (C) in the across the collective 300 ms post-stimulus period from the two conditions of interest (new in black vs. repeated in red). The reliability of this observation at the group-averaged level was then assessed at the individual level using a fitting procedure (see Material and Methods for further details; Figure S1).

*EEG results at T1 for novel objects versus repeated objects*

High-density EEG neuroimaging, combining brain microstate analysis with LAURA distributed linear source localizations, expanded the above mentioned fMRI results by revealing the temporal dynamics of the processing of new objects as compared to repeated objects at T1 (i.e., at the moment of hand-object interaction; Figure S2). The microstate analysis revealed seven time periods of stability: Map 1: 0-30 ms; Map 2: 32-60 ms; Map 3: 62-80 ms; Map 4: 82-120 ms; Map 5: 122-150 ms; Map 6: 152-320 ms; Map 7: 322-400 ms. Three topographies (Maps 3-5; Figure S2) were significantly present in the novel objects condition only. LAURA source estimations of this brain topography demonstrated similarities between our VEP and fMRI data by showing a dominant anterior cingulate activation for Map 2 (local maximum: 3, 41, -9; x, y, z mm Talairach coordinates); a dominant right-lateralized activation in the MTG/STS (local maximum: 57, -11, -5; x, y, z mm Talairach coordinates) for Map 4; and a right-lateralized activation including right IFG (local maximum: 46, 14, 25; x, y, z mm Talairach coordinates) for Map 5 (Table S3).

Analysis of global field power (GFP) revealed that Map 3’s temporal window (60-80 ms after hand-object interaction) was also characterized by a significant difference of GFP between novel objects (in red in Figure S2) and repeated objects (in white in Figure S2). Group-averaged data revealed a GFP peak at 64 ms for novel objects, although GFP for repeated objects was almost extinguished at the same time period. The reliability of this microstate at the group-averaged level was confirmed at the individual level using a Bonferroni corrected paired Ttest on GFP (Figure S2, color scale on the bottom row). More precisely, this paired Ttest revealed the following GFP differences: From 60-80 ms, with a peak at 64 ms (*P* < 0.05). Between 80-150 ms post hand-object interaction, no significant difference was observed in terms of GFP. Interestingly, after 150 ms post hand-object interaction, the reverse pattern (greater GFP for repeated objects than suppressed objects) was observed.
