# Supplementary figures and images for: Spatio-Temporal Dynamics of Human Intention Understanding in Temporo-Parietal Cortex: A Combined EEG/fMRI Repetition Suppression Paradigm
Source: PLoS One. 2009 Sep 11;4(9):e6962. doi: 10.1371/journal.pone.0006962 (PMC2736621; doi:10.1371/journal.pone.0006962)

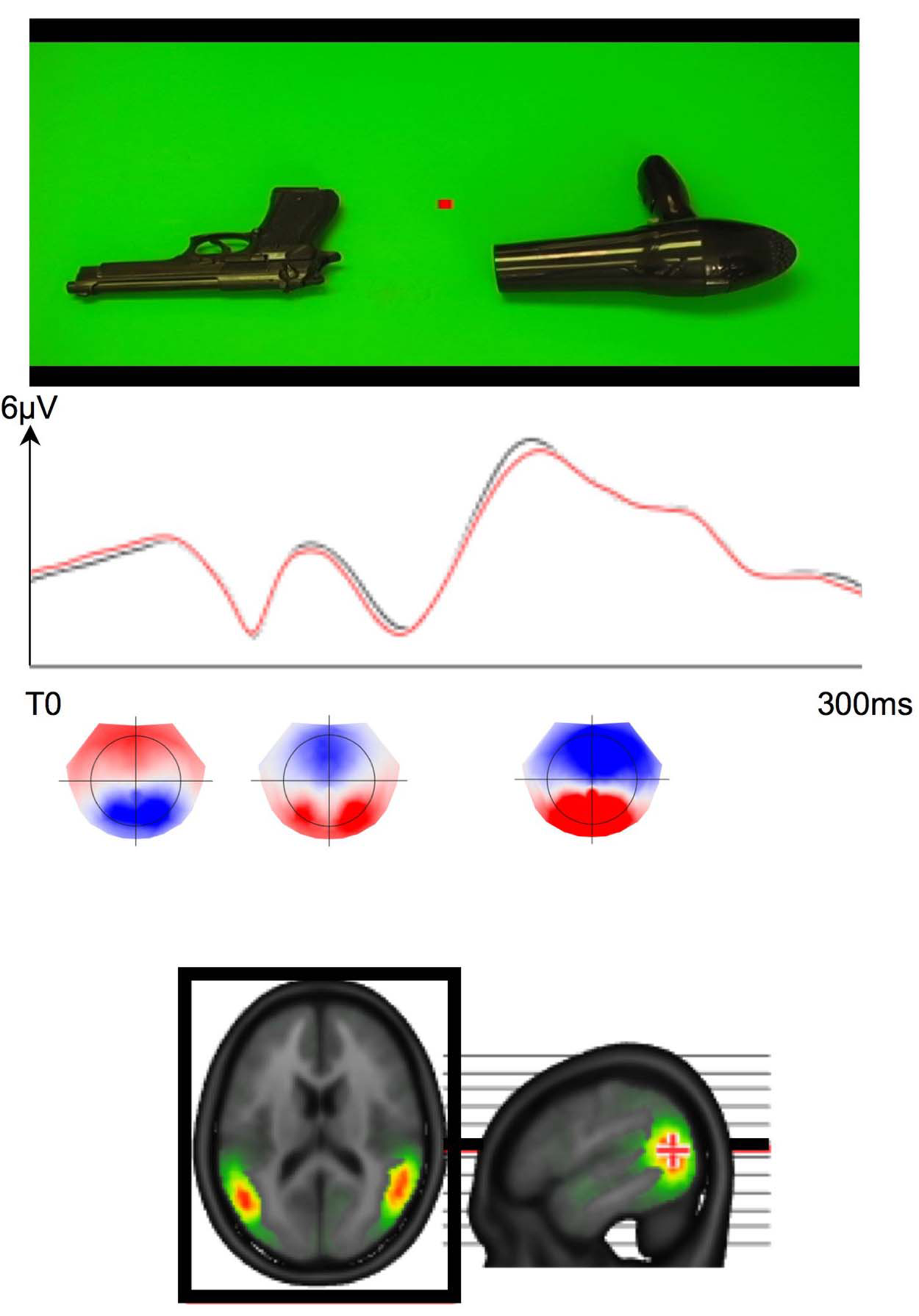

Supplement: Figure S1 — Electrical Neuroimaging results at T0. High-density EEG neuroimaging, combining brain microstate analysis with LAURA distributed linear source localizations at movie onset (T0; A), revealed archetypal VEPs components (e.g., C1, P1 and N1). The topographic pattern analysis of the global field power (B) identified three selective time periods of stable topography (C) in the across the collective 300 ms post-stimulus period from the two conditions of interest (new in black vs. repeated in red). All topographies are shown with the nasion upward and left scalp leftward. Intracranial brain generators as estimated with LAURA inverse solution over this period of time revealed a bilateral occipito-temporal activity as shown on axial plane. (9.70 MB TIF) [file pone.0006962.s002.tif]

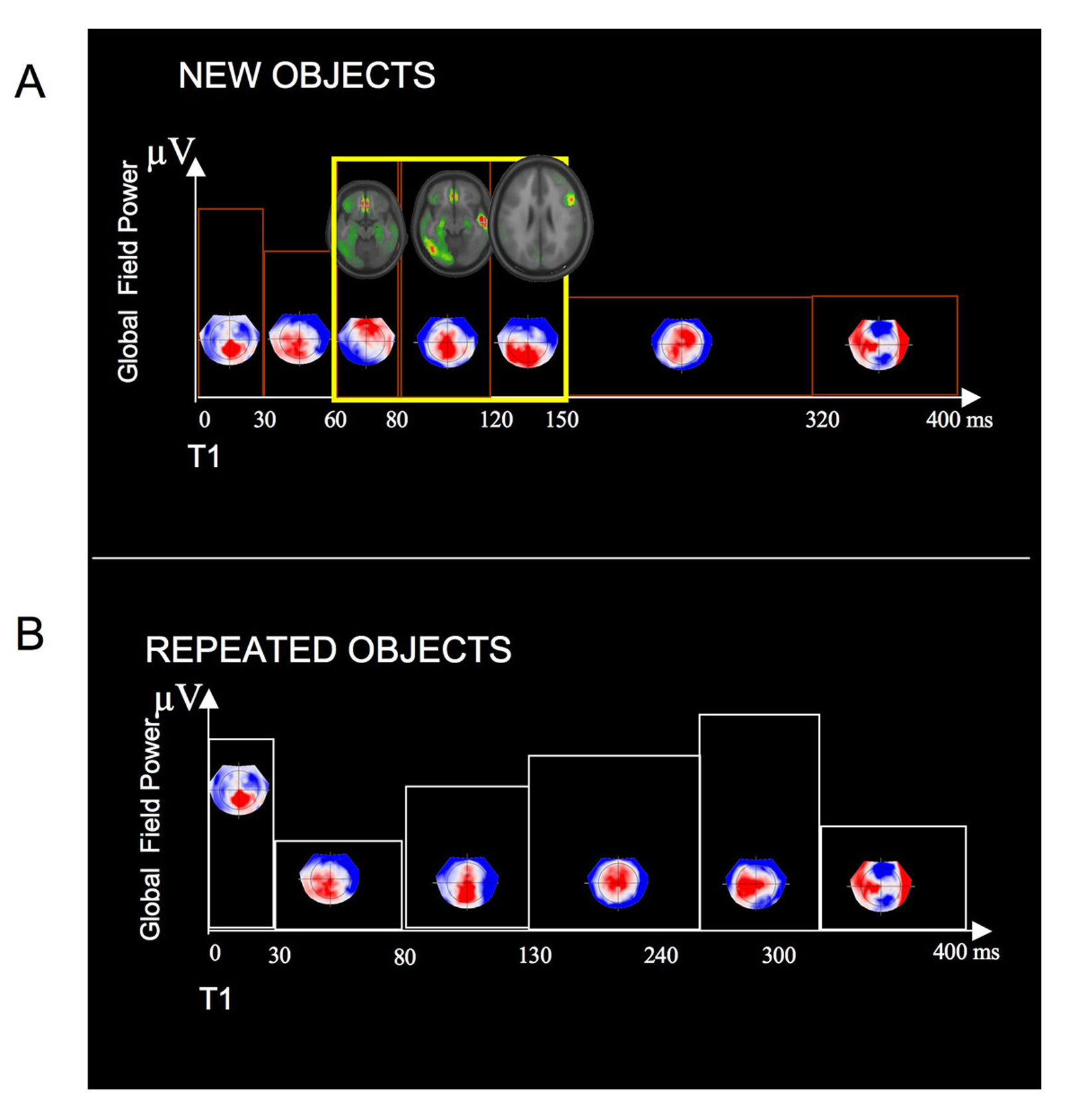

Supplement: Figure S2 — Electrical Neuroimaging results at T1 for new objects (A) and repeated objects (B). High-density EEG neuroimaging, combining brain microstate analysis with LAURA distributed linear source localizations applied at T1 (i.e., at the moment of hand-object interaction), revealed seven time periods of stability: Map 1: 0–30 ms; Map 2: 32–60 ms; Map 3: 62–80 ms; Map 4: 82–120 ms; Map 5: 122–150 ms; Map 6: 152–320 ms; Map 7: 322–400 ms. Three topographies (Maps 3–5) were significantly present in the novel objects condition only. Here, maps are represented on top of schematic representation of global field power. All topographies are shown with the nasion upward and left scalp leftward. LAURA source estimations of this brain topography demonstrated similarities between our VEP and fMRI data by showing a dominant anterior cingulate activation for Map 3 (local maximum: 3, 41, −9; x, y, z mm Talairach coordinates); a dominant right-lateralized activation in the right STS region and right aIPS (local maximum: 57, −11, −5; x, y, z mm Talairach coordinates) for Map 4; and a right-lateralized activation including right IFG and medial FG (local maximum: 46, 14, 25; x, y, z mm Talairach coordinates) for Map 5. Map 3's temporal window (62–80 ms after hand-object interaction) was also characterized by a significant difference of GFP between new objects and repeated objects. Group-averaged data revealed a GFP peak at 64 ms for new objects, although GFP for repeated objects was almost extinguished at the same time period. Between 80–150 ms post hand-object interaction, no significant difference was observed in terms of GFP. Interestingly, after 150 ms post hand-object interaction, the reverse pattern (greater GFP for repeated objects than suppressed objects) was observed. The reliability of this microstate at the group-averaged level was confirmed at the individual level using a Bonferroni corrected paired Ttest on GFP (P<0.05). Here, activations are represented on axial cross sections. [file pone.0006962.s003.tif]
