# Supplementary material for: Spatio-Temporal Dynamics of Human Intention Understanding in Temporo-Parietal Cortex: A Combined EEG/fMRI Repetition Suppression Paradigm
Source: PLoS One. 2009 Sep 11;4(9):e6962. doi: 10.1371/journal.pone.0006962 (PMC2736621; doi:10.1371/journal.pone.0006962)
Supplement: Table S1 — All ANOVAs had a level of significance set to 0.05. (0.04 MB DOC) [file pone.0006962.s004.doc]

**Table S1. Reaction times and accuracy: performance measures across types of object (new vs. repeated).**

|  | Reaction times |  | % Accuracy |  |
| --- | --- | --- | --- | --- |
|  | Mean (ms) | (S.E.) | Mean (%) | (S.E.) |
| Object type |  |  |  |  |
| **Gun** | **593** |  | **66** |  |
| New | 591 | (11.9) | 62 | (.40) |
| Repeated | 594 | (12.48) | 69 | (.28) |
| **Hairdryer** | **623** |  | **64** |  |
| New | 638 | (12.85) | 58 | (.45) |
| Repeated | 607 | (12.84) | 70 | (.33) |
| **New** | **615** |  | **60** |  |
| **Repeated** | **601** |  | **70** |  |
