# Supplementary material for: Spatio-Temporal Dynamics of Human Intention Understanding in Temporo-Parietal Cortex: A Combined EEG/fMRI Repetition Suppression Paradigm
Source: PLoS One. 2009 Sep 11;4(9):e6962. doi: 10.1371/journal.pone.0006962 (PMC2736621; doi:10.1371/journal.pone.0006962)
Supplement: Table S2 — Local maxima, with a Z values greater than 3 in each cluster, are provided in the table. Cluster size is in voxels. When the cluster encompasses more than one anatomical location, the localization given corresponds to the local maxima with the highest value. P<0.001 uncorrected. (0.03 MB DOC) [file pone.0006962.s005.doc]

**Table S2. Local maxima in MNI coordinates of cerebral activations peaks for new objects minus repeated objects.**

| Regions | M+> M- | | | | |
| --- | --- | --- | --- | --- | --- |
|  | X | y | z | Z | Cluster size |
| Right medial frontal gyrus | 48 | 17 | 27 | 3.98 | 45 |
| Left postcentral gyrus | -46 | -16 | 30 | 3.62 | 24 |
