# Supplementary material for: Spatio-Temporal Dynamics of Human Intention Understanding in Temporo-Parietal Cortex: A Combined EEG/fMRI Repetition Suppression Paradigm
Source: PLoS One. 2009 Sep 11;4(9):e6962. doi: 10.1371/journal.pone.0006962 (PMC2736621; doi:10.1371/journal.pone.0006962)
Supplement: Table S3 — Local maxima (in Talairach coordinates) of periods of brain stability (i.e., microstates) are provided in the table. Asterisk indicates the time period that is significantly present in the novel object condition in comparison with the repeated object condition. P<0.05. (0.04 MB DOC) [file pone.0006962.s006.doc]

**Table S3. Local maxima of current source density obtained from LAURA for novel and repeated objects, respectively.**

| Microstates | Brain region labels | Brain coordinates | | |
| --- | --- | --- | --- | --- |
|  |  | x | Y | z |
| 1 | Left occipito-parietal | -15 | -78 | 35 |
| 2 | Left temporal | -59 | -31 | -2 |
| **3** | Anterior cingulate | -3 | -45 | 1 |
| **4** | Right temporal | 58 | -11 | -9 |
| **5*** | Right IFG | 47 | 20 | 25 |
| 6 | Right temporal | 58 | -11 | -9 |
| 7 | Right temporal | 54 | -1 | -13 |
